# Is There a Role for Epigenetic Therapies in Modulating DNA Damage Repair Pathways to Enhance Chemotherapy and Overcome Drug Resistance?

**DOI:** 10.3390/cancers14061533

**Published:** 2022-03-16

**Authors:** Ian Matthew Garner, Robert Brown

**Affiliations:** Department of Surgery and Cancer, Imperial College London, London W12 0NN, UK; i.garner@imperial.ac.uk

**Keywords:** DNA repair, epigenetics, cancer, DNA methylation

## Abstract

**Simple Summary:**

Epigenetic changes in a cell’s genome can cause the inactivation of genes and affect how tumours respond to chemotherapy. These epigenetic changes do not alter the DNA sequence and small molecule inhibitors of maintenance of the epigenetic state can change the sensitivity of a tumour to chemotherapy. Epigenetic therapies are being clinically evaluated as single agents or in combination with chemotherapies, including those that induce DNA damage. DNA damage induced by chemotherapy may be repaired and the cell either survives or alternatively cell death pathways are engaged and the cell is eliminated. Epigenetic silencing of gene expression during tumour development may lead to sensitivity of the tumour to chemotherapy if DNA repair of the DNA lesion is inactivated, or to chemotherapy resistance if cell death responses are inactivated. This illustrates the clinical challenge of using epigenetic therapies in combination with chemotherapy. Will such epigenetic drugs confer chemosensitivity and increased efficacy of chemotherapy, or induce resistance? Is there an effect of epigenetic therapies on normal cell chemo-sensitivity leading to increased toxicity? This review will illustrate the clinical challenges in combination studies of epigenetic therapies with chemotherapy and discuss future perspectives on addressing these challenges.

**Abstract:**

Epigenetic therapies describe drug molecules such as DNA methyltransferase, histone methyltransferase and histone acetylase/deacetylase inhibitors, which target epigenetic mechanisms such as DNA methylation and histone modifications. Many DNA damage response (DDR) genes are epigenetically regulated in cancer leading to transcriptional silencing and the loss of DNA repair capacity. Epigenetic marks at DDR genes, such as DNA methylation at gene promoters, have the potential to be used as stratification biomarkers, identifying which patients may benefit from particular chemotherapy treatments. For genes such as *MGMT* and *BRCA1*, promoter DNA methylation is associated with chemosensitivity to alkylating agents and platinum coordination complexes, respectively, and they have use as biomarkers directing patient treatment options. In contrast to epigenetic change leading to chemosensitivity, DNA methylation of DDR genes involved in engaging cell death responses, such as *MLH1*, are associated with chemoresistance. This contrasting functional effect of epigenetic modification on chemosensitivity raises challenges in using DNA-demethylating agents, and other epigenetic approaches, to sensitise tumours to DNA-damaging chemotherapies and molecularly targeted agents. Demethylation of *MGMT/BRCA1* could lead to drug resistance whereas demethylation of *MLH1* could sensitise cells to chemotherapy. Patient selection based on a solid understanding of the disease pathway will be one means to tackle these challenges. The role of epigenetic modification of DDR genes during tumour development, such as causing a mutator phenotype, has different selective pressures and outcomes compared to epigenetic adaptation during treatment. The prevention of epigenetic adaptation during the acquisition of drug resistance will be a potential strategy to improve the treatment of patients using epigenetic therapies.

## 1. Deoxyribonucleic Acid (DNA) Damage Response (DDR) Genes Are Epigenetically Regulated in Cancer, Affecting Chemosensitivity

Epigenetic modifications, such as gene promoter DNA hypermethylation, and subsequent changes in gene expression of DDR-associated genes, lead to a loss of DNA repair capacity and have been demonstrated in a variety of tumours and cell line models [1,2]. The loss of DNA repair activity in tumours may lead to chemosensitivity to DNA-damaging cytotoxic chemotherapy. One of the paradigms demonstrating the clinical relevance of epigenetic mechanisms involved in chemosensitivity is highlighted in glioblastoma. DNA methylation of *MGMT*, the DNA repair gene encoding O-6-methylguanine-DNA methyltransferase, leads to the loss of *MGMT* expression [1]. This leads to reduced DNA damage repair and subsequently increased sensitivity of cells to the alkylating agent, temozolomide [3]. Early clinical studies showed that glioma patients treated with temozolomide and radiotherapy and with a methylated *MGMT* gene promoter have a survival benefit compared to only radiotherapy. Patients with unmethylated *MGMT* promoters showed no statistically significant difference in survival [4]. Prospective randomised trials of glioblastoma patients for radiotherapy versus alkylating agent chemotherapy have demonstrated DNA methylation of *MGMT* is a clinically useful predictive biomarker to stratify patients, rather than just prognostic [3,5,6].

A second paradigm is the epigenetic alterations of homology recombination DNA repair (HR)-associated genes including breast cancer type 2 susceptibility proteins 1 and 2 (*BRCA1/2*) [7,8]. *BRCA1* is frequently methylated in high-grade serous ovarian cancer (HGSOC) and can lead to HR-deficiency (HRD) which is associated with increased patient survival following platinum-based chemotherapy compared to patients with HR proficient tumours [8]. Sensitivity to platinum-based chemotherapy exploits HRD in HGSOC by introducing double-stranded breaks in DNA, leading to genomic instability and cell apoptosis [9]. However, non-homologous end joining (NHEJ) and base excision repair (BER) pathways, which require Poly (ADP-ribose) polymerase (PARP), can still be utilised to repair the damaged DNA. PARP inhibitors (PARPi) induce synthetic lethality in HR-deficient tumours by disrupting multiple DNA repair pathways simultaneously [10]. In breast and ovarian cancer, *BRCA1/2* status is clinically useful to predict sensitivity to platinum-based chemotherapy and PARPi [11,12]. Furthermore, patients with *BRCA1* methylated and HRD HGSOC have better prognosis than unmethylated HR proficient tumours [13].

In both the above paradigms, epigenetic regulation by DNA methylation during tumour development, prior to chemotherapy, leads to inactivation of DNA repair activity and drug sensitivity. However, epigenetic mechanisms have also been proposed as important drivers of acquired drug resistance adaptation during chemotherapy. This leads to increased epigenetic silencing in tumours at relapse compared to primary presentation [14]. For instance, loss of DNA mismatch repair (MMR) due to DNA methylation at the MutL Homolog 1 (*MLH1*) gene promoter has been associated with resistance to alkylating agents such as temozolomide and crosslinking agents such as cisplatin [15,16,17]. The presence of functional MMR has been proposed to lead to cell death due to futile repair cycles, generation of double-strand DNA breaks and engagement of apoptosis [18]. Thus, the absence of MMR leads to loss of engagement of cell death pathways by DDR pathways leading to drug resistance. In another example of epigenetic adaptation following chemotherapy, while sensitivity to PARP inhibitors of HGSOC is associated with DNA methylation at *BRCA1*, tumours recurring following chemotherapy restore BRCA1 expression associated with reduced DNA methylation. This supports a key role for DNA methylation changes during the acquisition of PARPi resistance [19]. *FANCF*, another DDR-associated gene closely linked to BRCA genes, is often methylated in several different cancer types including testicular [20], head and neck [21], lung [21], cervical [22] and ovarian [23,24]. Methylation of the *FANCF* promoter in ovarian cancer has been linked to platinum sensitivity, whereas demethylation of *FANCF* has been associated with platinum resistance and often occurs after platinum chemotherapy [24,25].

**Figure 1:**
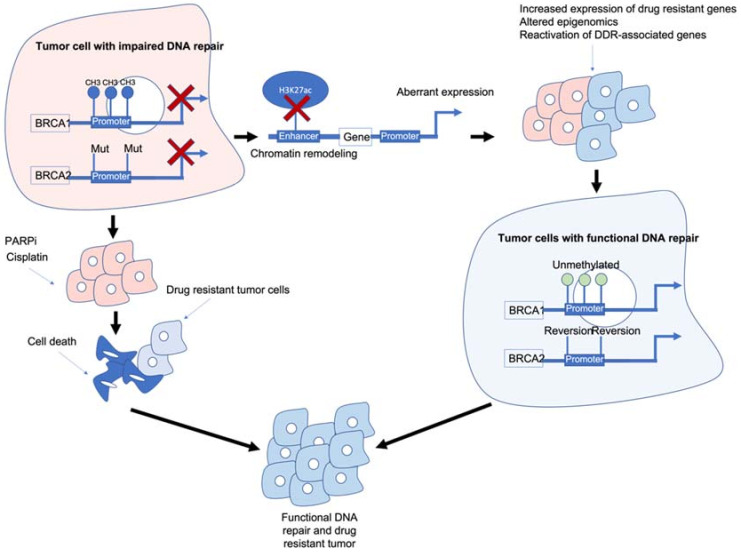
Epigenetic interactions with breast cancer type 1/2 susceptibility (*BRCA1/2*) genes and drug resistance. *BRCA1/2* deficiency caused by methylation and/or mutations results in impaired DNA repair and often sensitivity to poly (ADP-ribose) polymerase inhibitors (PARPi)/platinum-based chemotherapies. *BRCA1/2* deficiency can also modulate chromatin accessibility and enhancers of drug-resistant and/or DNA damage response (DDR)-associated genes. Reactivation of *BRCA1/2* either by demethylation or reversion mutations can result in tumour cells with functional DDR. Ultimately, these mechanisms result in tumour cells that are drug resistant, have functional DDR and no longer respond to previous PARPi/platinum chemotherapy.

Whilst aberrant methylation of DDR genes has been shown in multiple cancers, other epigenetic mechanisms such as histone modifications at genomic regulatory regions, including enhancers and super enhancers, may also play an important role in response to chemotherapy. Studies show modest benefits of temozolmide treatment in patients with methylated *MGMT* in colorectal cancer and patient-derived xenograft (PDX) models of glioblastoma show high expression of *MGMT* linked to active enhancers, despite promoter methylation [26]. This suggests different epigenetic mechanisms are able to dynamically regulate gene expression. Furthermore, DDR-associated genes associated with drug response may themselves regulate the epigenetic landscape. *BRCA1* mutations in breast cancer epithelial cells lead to the loss of H3K27ac at super enhancers and impair enhancer–promoter lopping [27], whereas *BRCA2* depletion has been linked to chromatin remodelling [28]. MMR inactivation via *MLH1* mutations has been shown to activate enhancers of genes associated with growth in colorectal cancer and may activate enhancers of genes associated with drug-resistance [29]. These observations highlight the potential interplay of different epigenetic mechanisms and DDR-associated genes in relation to chemosensitivity (Figure 1).

*BRCA1/2* deficiency can impair the HR pathway leading to increased sensitivity to DNA-damaging agents such as platinum chemotherapy and PARPi. However, *BRCA1/2* deficiency can also lead to chromatin remodelling and reduced H3K27ac at regulatory regions such as enhancers/super-enhancers, leading to the aberrant expression of genes including those associated with drug resistance/DDR-associated genes. Reactivation of *BRCA1/2* and/or other DDR-associated genes can result in tumour cells which are drug resistant and have functional DNA damage repair.

## 2. Can Epigenetic Therapies Reverse Epigenetically Driven Drug Resistance?

The clinically relevant examples above demonstrate how promoter DNA methylation may confer tumour chemosensitivity or chemoresistance. However, there is a wide spectrum of DDR genes whose epigenetic regulation can influence chemosensitivity [2]. Epigenetic therapies such as DNA-demethylating agents and inhibitors of maintenance of histone post-translational modification (for instance, histone deacetylase inhibitors) are now registered for clinical use, particularly in haematological malignancies. Furthermore, they remain the focus of many clinical trials in epithelial cancers [30]. However, careful patient selection will be key to demonstrating clinical efficacy of epigenetic therapies, especially when used in combination with other therapies.

This is exemplified in early clinical trials of DNA-demethylating agents in HGSOC. In cell line models, the loss of MMR due to *MLH1* methylation results in failure to engage apoptotic responses and resistance to platinum coordination complexes and alkylating agents which can be reversed by DNA-demethylating agents such as 5-azacytidine and its derivatives [31]. DNA methylation has been used as a pharmacodynamic marker in surrogate tissues, such as blood, to demonstrate biological activity and guide the scheduling of combination studies with other therapies [32]. There have been two randomised phase II studies of DNA-demethylating agents and carboplatin in HGSOC with contrasting outcomes [33,34]. One study was closed early due to unacceptable toxicity and lack of efficacy of the combination compared to single-agent carboplatin [33]. The other trial showed an improvement in the 6-month progression-free survival of patients treated with the combination. However, this trial did not show statistically significant superiority for the primary endpoint of progression-free survival, potentially due to being statistically underpowered for the latter endpoint [34]. Both studies explored the combination of carboplatin with a DNA-demethylating agent during second-line chemotherapy. Glasspool et al., recruited partially platinum-sensitive patients recurring 6–12 months following the initial response to platinum-based chemotherapy while Oza et al., recruited women with recurrence within 6 months of the last platinum-containing regimen. It is possible that partially platinum sensitive patients may have a different proportion of women with tumours who are sensitive due to the methylation of HR genes, such as *BRCA1*, and for whom a demethylating agent may have an adverse effect. Neither study selected patient recruitment based on the methylation status of the patients’ tumours.

As previously mentioned, *BRCA1* is frequently methylated in high-grade serous ovarian cancer and associated with increased patient survival following platinum-based and PARP inhibitor chemotherapy compared to patients with HR-proficient tumours. BRCA1/2 deficiency remains the strongest predictor of PARPi sensitivity [35] although abrogation of other key HR genes including FA Complementation Group A (*FANCA*) [36], DNA Repair Protein RAD51 homolog 1 (*RAD51*) [37], X-ray Repair Cross Complementing 2 (*XRCC2*) and X-ray Repair Cross Complementing 3 (*XRCC3*) [38] and DNA Polymerase Delta 4 (*POLD4*) [39] have been linked to platinum and/or PARPi responses. Furthermore, not all BRCA mutant tumours are HR deficient and many HR-proficient tumours can initially respond well to PARPi [40,41,42,43,44] which has been attributed to the involvement of PARP in other non-DDR associated mechanisms including chromatin remodelling [45]. Unfortunately, as with platinum-based chemotherapy, primary and acquired resistance to PARPi is common [35,40,46,47]. Reversion mutations can restore the function of HR-associated genes frequently mutated in HGSOC, including *BRCA1/2* [48,49,50,51] and *RAD51C/D* [52]. Epigenetic mechanisms including histone modifications may also contribute to PARPi resistance although the exact mechanisms remain poorly understood [12,52,53,54,55,56,57].

Histone methylation has been linked to PARP inhibitor sensitivity in multiple cancers [58]. Enhancer of Zeste Homolog 2 (*EZH2*) and Euchromatic Histone Lysine Methyltransferase 2 (*EHMT2*) both maintain repressive H3K27 and H3K9 methylation histone marks, respectively, and are frequently overexpressed in cancer [59]. The inhibition of *EZH2* alone has previously been linked to reducing the expression of multiple genes associated with DDR pathways in multiple cancers including prostate [60] and ovarian [61]. Furthermore, *EZH2* inhibition has been shown to sensitise breast cancer cells to PARPi [58,61] and PARPi can regulate *EZH2* expression [62] via PARPylation. *EHMT2* has been linked with directly recruiting HR-associated factors, including *BRCA1* to promote DNA damage repair [63]. The inhibition of *EHMT2* promotes increased DNA damage and altered cell cycle regulation [64] and PARPi resistant cells treated with an *EHMT1/2* inhibitor show significantly altered gene expression changes enriched in pro-survival pathways including, phosphatidylinositol 3 kinase(PI3K), protein kinase B (AKT) and mammalian target of rapamycin (mTOR) [64]. In *BRCA1*-depleted SUM149 breast cancer cells and PDX models, treatment with an EZH2i and PARPi reduced tumour growth more than single PARPi treatment; however, this effect was not seen in a *BRCA2*-depleted mouse model of breast cancer [65]. *EZH2* inhibition alone may not be sufficient to modulate chromatin conformation [66] therefore dual *EZH2/EHMT2* inhibitors in combination with PARPi would perhaps be of future interest. A clear theme regarding epigenetic therapies in combination with chemotherapy and their ability to modulate epigenetically driven drug resistance is that any future combination therapies must have clear stratification markers. The global epigenetic and mutational profile of DDR-associated genes must also be considered in order to maximise the positive outcomes for patients. A summary of key DDR genes associated with drug resistance and/or regulated by epigenetic mechanisms are shown in Table 1.

## 3. Epigenetic Changes in Normal Tissue following Chemotherapy

The observations of increased DNA methylation at gene promoters in drug resistant tumours and cell line models following chemotherapy treatment could be due to the selection of cells epigenetically silenced that are present in the tumour before chemotherapy. Alternatively, DNA damage induced by the chemotherapy may be causing methylation changes. DNA damage such as platinum-induced and DNA double-strand breaks are recognized by DNA mismatch repair proteins [67]. These bind and recruit the DNA methylating enzyme encoded by the *DNMT1* gene, resulting in aberrant DNA methylation [68,69,70,71]. At the time of relapse following platinum-based chemotherapy, changes in methylation at specific CpG sites in blood DNA are observed which mirror changes occurring in tumour DNA at relapse in ovarian cancer patients [72]. These changes can predict clinical outcome and identify patients with better overall survival. In contrast, blood samples taken at presentation prior to treatment show no association between methylation and survival. DNA methylation at specific CpGs in blood has been associated with environmental exposures including smoking and alcohol consumption [73,74]. Smoking-induced methylation changes at the *ARRH* gene have been associated with aberrant *ARRH* transcription in lung epithelial cells [60] and mediate the risk of developing lung cancer [75]. In 2020, it was estimated that 4.1% of all cancers can be attributed to alcohol consumption [76]. A 144 CpG DNA methylation signature has been used to identify heavy alcohol consumption in whole blood samples [77]. Whilst the functional consequences of methylation changes in blood remain unknown, they may be acting as a surrogate markers for changes in a more relevant tissue. Similarly, epigenetic changes occurring in normal tissues due to DNA-damaging agents such as chemotherapy can have long-term consequences for secondary tumours or altering immune responses [78].

## 4. Challenges and Future Prospects

Epigenetic drugs including inhibitors of DNA methyltransferases, histone methyltransferases and histone acetylases/deacetylases can all affect genome-wide transcription. Therefore, they may be a doubled-edged sword by globally activating or inhibiting DDR-associated genes or tumour suppressors/oncogenes or activating pathways which have negative impacts on other drug agents. Drugs targeting specific markers such as PARP inhibitors may also affect the global expression of genes. *PARP1* can modulate chromatin confirmation and epigenetic regulation [79,80]; therefore, PARPi may cause off-target effects which are not currently clear. Reactions to PARPi have been documented with evidence suggesting that whilst patients with germline BRCA mutations may respond well to PARPi [10,81], their risk of developing other diseases including myelodysplastic syndrome and acute myeloid leukemia is increased [82]. HR or BRCA restoration may also negate any benefits of PARPi use [83,84], further highlighting the need to time treatment and develop biomarkers to predict not only the response but also the probability of HR restoration during treatment.

Epigenetic mechanisms underpin immune evasion in cancer. In pre-clinical models, the inhibition of *DNMT1* and *EZH2* improves the efficacy of immune checkpoint inhibitor therapy, slows tumour progression and increases TH1-cytokine production [85]. A key mechanism involves viral defence responses. *DNMT1* inhibition triggers cytosolic sensing of double-stranded RNA (dsRNA), thereby inducing type I interferon responses. Many of the dsRNA species arise from the reactivation of heavily methylated endogenous retroviruses [86]. These data suggest that combining an immune checkpoint inhibitor with a DNA-demethylating agent may increase response rates. In 2017, the FDA approved the use of pembrolizumab for the treatment of malignancies with microsatellite instability (MSI-H) or mismatch repair deficiency (dMMR). It is hypothesised that a higher somatic mutational load leads to increased presentation of neoepitopes thus mediating immunotherapy responses in MSI-H tumours. In colorectal cancer, MSI-H tumours evolve gene expression changes (immunoediting) that may confer resistance to recognition by the immune system and hence resistance to pembrolizumab, despite high mutational load and frequent lymphocytic infiltration. Furthermore, these tumours also have genomic and DNA methylation events associated with activated WNT signalling and T-cell exclusion [87]. Tumours with dMMR during treatment may be resistant to checkpoint inhibitors due to epigenetic mechanisms driving immunoediting. Hence, the combination of pembrolizumab and a DNA-demethylating agent may be particularly beneficial to patients that have lost the expression of MMR genes due to DNA methylation.

The selection of patients most likely to benefit will be crucial for effective clinical evaluation of epigenetic therapies given alone or in combination with DNA-damaging agents. While many potential predictive biomarkers of drug sensitivity have been proposed in preclinical models, few have been tested in clinical trials of epigenetic therapies and none yet have been used as companion biomarkers during their clinical use. This is partly because most biomarkers identified from preclinical studies have been tested as prognostic rather than predictive biomarkers in clinical studies. This is partly due to the relative ease of doing prognostic studies that need considerably fewer patient samples. To exemplify this, a prognostic study in ovarian cancer to detect a hazard ratio of 2 at 80% power at *p* = 0.05 would require approximately 110 tumour samples. A predictive study to compare a biomarker between two treatment arms at similar statistical power would require over 1200 tumours. The challenges in identifying predictive biomarkers have previously been reviewed and potential roadmaps for their evaluation developed [88,89]. However, most clinical trial designs include the predictive biomarker late on in the clinical evaluation pathway. Perhaps the identification of molecular subgroups based on a biological understanding of the mechanism of action of an epigenetic therapy needs to be included earlier in the pathway, for instance, by selecting patients based on an epigenomic subtype of DDR genes, rather than histological subtype in phase II studies of combinations with DNA-damaging cytotoxics.

Trial designs which incorporate stratification biomarker evaluation will be particularly important in combining DNA-damaging therapies with epigenetic agents. This is clearly exemplified with DNA-demethylating agents where the demethylation of genes such as *MGMT* and *BRCA1* could lead to resistance to cytotoxics and treatment failure, while demethylation of MMR genes and other pro-apoptotic genes could lead to increased efficacy in response to cytotoxics inducing DNA damage. The clinical timing of epigenetic intervention will also be important. Tumours at initial presentation will have genetic and epigenetic changes as a consequence of tumorigenesis where loss of DNA damage responses and genomic instability are important drivers. In contrast, tumours at the time of recurrence will have changes as a consequence of tumour adaptation during chemotherapy as well as tumorigenic evolution. Furthermore, DDR-associated genes can be dynamically regulated by more than one epigenetic mechanism. *MGMT* expression can still be high despite promoter DNA hypermethylation due to histone acetylation at active enhancer regions [26]. Therefore, one can envisage a scenario where a patient who has a methylated gene associated with drug resistance may appear as a candidate who could benefit from treatment with a DNMT inhibitor, yet this may fail due to histone modification having a greater effect on expression. Another particularly interesting speculation is whether epigenetic therapies could be used during remission as a way to prevent the emergence of epigenetic adaptation and resistance. Delaying recurrence or maintaining tumours in a chemotherapy sensitive state would have major effects on the overall survival of cancer patients. However, this assumes that epigenetic therapies do not have adverse effects on promoting tumour progression or normal cell toxicity.

## 5. Conclusions

Epigenetic marks play a key role in drug sensitivity/resistance. Epigenetic therapies, including DMNT inhibitors which target DNA methylation, HKMT inhibitors which target histone methylation and HDAC inhibitors which target histone deacetylases, could play an important role in enhancing chemotherapy by modifying epigenetic marks associated with resistance. Several DDR-associated genes can be regulated by epigenetic mechanisms such as DNA methylation, with recent evidence showing DDR-associated gene expression can be controlled by multiple epigenetic mechanisms including histone methylation and acetylation. Furthermore, specific DDR-associated genes inducing *BRCA1/2* can directly modulate epigenetic mechanisms such as chromatin conformation [28] and enhancer activity [27,90] providing a strong rationale to combine epigenetic therapies with current chemotherapy in order to circumvent acquired resistance and enhance chemotherapy outcomes. The stratification of patients based on the mutation status of key DDR-associated genes, such as *BRCA1/2* and *MGMT*, will require careful consideration with respect to their epigenetic state in order to predict a robust response to chemotherapy.

## Figures and Tables

**Table 1:** Summary of DDR-associated genes, how they can be epigenetically regulated and their involvement in drug response.

Gene	Symbol	Summary	Reference Number
O6-Methylguanine-DNA Methyltransferase	*MGMT*	Methylated associated with increased sensitivity to temozolomide. Enhancer region associated with increased expression and resistance to temozolomide.	[1,3,5,6,26]
Breast Cancer type 1 susceptibility protein	*BRCA1*	Methylated associated with sensitivity to PARPi/platinum and loss of H3K27ac at enhancer regions.	[7,8,11,12,27,35]
Breast Cancer type 2 susceptibility protein	*BRCA2*	Deficiency causes chromatin conformation changes and increased sensitivity to PARPi/platinum.	[7,8,11,12,28]
MutL Homolog 1	*MLH1*	Unmethylated associated with temozolomide/platinum resistance and loss of MMR.	[15,16,17,31]
FA Complementation Group F	*FANCF*	Methylation associated with sensitivity to platinum, unmethylated associated with platinum resistance.	[24,25]
FA Complementation Group A	*FANCA*	Germline mutation associated with increased sensitivity to DNA damaging agents.	[36]
DNA Repair Protein RAD51 homolog 1	*RAD51*	High expression associated with platinum resistance.	[37]
X-ray Repair Cross Complementing 2	*XRCC2*	Low expression associated with sensitivity to PARPi.	[38]
X-ray Repair Cross Complementing 3	*XRCC3*	Low expression associated with sensitivity to PARPi.	[38]
DNA Polymerase Delta 4	*POLD4*	Low expression associated with sensitivity to PARPi/platinum.	[39]
RAD51 Paralog C	*RAD51C*	Reversion mutations associated with increased resistance to PARPi.	[52]
RAD51 Paralog D	*RAD51D*	Reversion mutations associated with increased resistance to PARPi.	[52]
Euchromatic Histone Lysine Methyltransferase 2	*EHMT2*	Maintains repressive H3K9 methylation marks. Recruits HR-associated factors, Inhibition of EHMT2 promotes DNA damage.	[58,59,63,64,66]
Enhancer of Zeste Homolog 2	*EZH2*	Maintains repressive H3K27 methylation marks. Controls expression of multiple DDR-associated genes. Inhibition of EZH2 sensitises cells to PARPi.	[58,59,60,61,66]

## Data Availability

No new data were created or analyzed in this study. Data sharing is not applicable to this article.
